# Data on energy-band-gap characteristics of composite nanoparticles obtained by modification of the amorphous potassium polytitanate in aqueous solutions of transition metal salts

**DOI:** 10.1016/j.dib.2016.04.015

**Published:** 2016-04-11

**Authors:** D.A. Zimnyakov, A.V. Sevrugin, S.A. Yuvchenko, F.S. Fedorov, E.V. Tretyachenko, M.A. Vikulova, D.S. Kovaleva, E.Y. Krugova, A.V. Gorokhovsky

**Affiliations:** Yuri Gagarin State Technical University of Saratov, 77 Politechnicheskaya Street, 410054 Saratov, Russia

## Abstract

Here we present the data on the energy-band-gap characteristics of composite nanoparticles produced by modification of the amorphous potassium polytitanate in aqueous solutions of different transition metal salts. Band gap characteristics are investigated using diffuse reflection spectra of the obtained powders. Calculated logarithmic derivative quantity of the Kubelka–Munk function reveals a presence of local maxima in the regions 0.5–1.5 eV and 1.6–3.0 eV which correspond to band gap values of the investigated materials. The values might be related to the constituents of the composite nanoparticles and intermediate products of their chemical interaction.

**Specifications Table**

**Table:** 

Subject area	*Materials science*
More specific subject area	*Physical properties*
Type of data	*Table, figures*
How data was acquired	*Energy-Dispersive X-ray Fluorescence Spectrometer BRA-135F UV–vis spectrophotometer ShimadzuUV2550PC equipped with an ISR-220 integrating sphere*
Data format	*Analyzed*
Experimental factors	*The spectra of diffuse optical reflection from the compacted powders of the amorphous potassium polytitanate modified in aqueous solutions of transition metal salts were recorded in a 200–1200* *nm wavelength range and were used to estimate band gap for the constituents of the composite nanoparticles and intermediate products of their chemical interaction.*
Experimental features	*Composite nanoparticles were produced by modification of amorphous potassium polytitanates in aqueous solution of copper, iron, nickel, zinc, chromium and cobalt sulfates.*
Data source location	*Department of Chemistry, Yuri Gagarin State Technical University of Saratov, Russia*
Data accessibility	*Data are available with this paper*

**Value of the data**

● The data reveals a complex configuration of electronic zones for the products obtained by soft chemistry methods such as modification of layered potassium polytitanate particles in aqueous solutions of transition metal salts.
● This data attributes band gap values of the modified potassium polytitanates to the particular transition metal salt chosen for the modification.
● The data can be applied to select the materials with optimal electron zones configuration for their application in different electronic devices.

**Data**

The dataset consists of energy-band-gap characteristics of the potassium polytitanate nanoparticles modified in the aqueous solutions of transition metal salts of organic acids including Cu(II), Fe(II), Co(II) oxalates, Zn formate, as well as Cr(II) and Ni(II) acetates at pH=5–6. The data are obtained using modified Kubelka–Munk spectral method which allows recognizing a presence of particles with different energy-band-gap characteristics.

## 1 Experimental design, materials and methods

The data on energy-band-gap characteristics are obtained using spectral method described in our previous work [1] and based on modified Kubelka–Munk approach [[2], [3]]. This method allows obtaining configurations of electronic zones in the mixtures of particles, including nanoparticles, characterized with varied structure and composition such as amorphous and quasiamorphous objects. The following operations have been done to obtain the energy-band-gap datasets for the materials investigated. The so-called Tauc plots of {*α*(*E*)*·E*}^*n*^=*f*(*E*) have been constructed, where *E* is the photon energy and *α*(*E*) *is* the absorption coefficient replaced for optically dense systems by the Kubelka–Munk function *F*(*E*). The *F*(*E*) values is determined in accordance with [4], [5] from the spectral dependence of diffuse reflection coefficient *R*(*E*) by the Eq. (1):

(1) $$F\left(E\right)=\left(1-R^{2}\right)/2R\sim \alpha .$$

Determining local maxima of a logarithmic derivative quantity defined as Δ(*E*)*=d*{*ln*[*F*(*E*)*E*]}/*dE* allows reliable estimation of *E*_*g*_ taking into account that the inclusion of the interband absorption at *E*≥*E*_*g*_ must be accompanied by the appearance of the local maximum in Δ(*E*) corresponding to the conditions of *E*=*E*_*g*_. Discrepancies between *E*_*g*_ estimations obtained using the traditional Tauc plot extrapolation method and the proposed approach do not exceed 0.02 eV. If a system consists of several kinds of particles, each type of particles characterized with similar electron structure can be described as related to the specific local maximum.

The composite nanoparticles obtained by chemical modification of the amorphous particles of potassium polytitanate (PPT) in the aqueous solutions of copper, iron, nickel, zinc, chromium and cobalt salts of organic acids are investigated using the abovementioned experimental method.

## 2 Material preparation

The amorphous potassium polytitanate powder (trade-mark PPT-4, Nanocomposite Ltd., Russia), consisted of platy nanoparticles with a mean diameter of 273±125 nm and a thickness of 5–15 nm was utilized in the experiments as raw material. The TiO_2_:K_2_O molar ratio of the powder corresponded to 4.05:1.00. The PPT powder was modified according to the following route [5], [6], [7]: 15 g of powder was put in a glass which then was filled with 100 ml of 10^−3^ M aqueous solution of the corresponding transition metal salts. The obtained suspension was stirred for 8 h and then the obtained product was separated by centrifugation and dried at 60 °C for 4 h. The chemical composition of the prepared powders was determined in the oxide form using an energy-dispersive X-ray fluorescence spectrometer and reported in Table 1.

Further, modified PPT powders (PPTM) were compacted to density 2.25±0.15 g/cm^3^ in disks of 6 mm in diameter and 2.0±0.1 mm of thickness and used to investigate their optical reflection spectra.

## 3 Spectral data

**Fig. 1:**
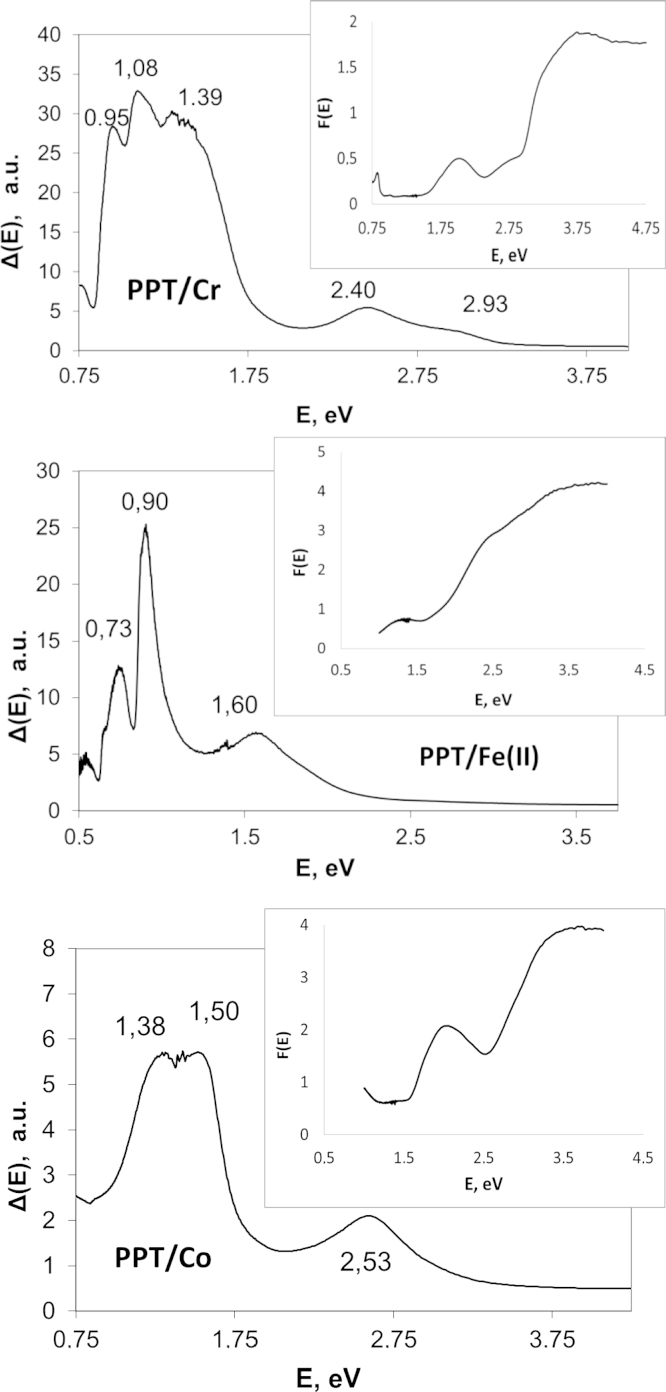
Plots of Δ(*E*) versus band gap energy and (inset) the corresponding spectra of the Kubelka–Munk function *F*(*E*) for the potassium polytitanates modified in aqueous solutions of cobalt, iron and chromium salts.

**Fig. 2:**
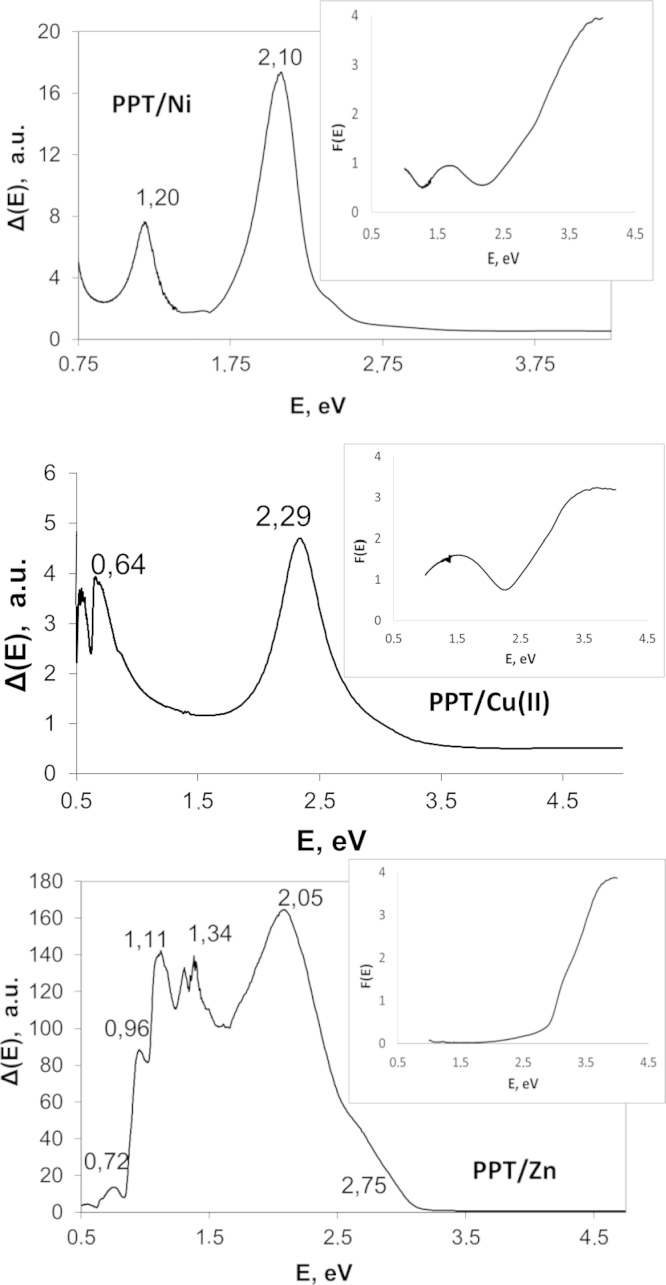
Plots of Δ(*E*) versus band gap energy and (inset) the corresponding spectra of the Kubelka–Munk function *F*(*E*) for the potassium polytitanates modified in aqueous solutions of copper, nickel and zinc salts.

The measured diffuse reflection spectra has been processed to obtain the Kubelka–Munk functions *F*(*E*) and corresponding Δ(*E*) functions for different PPT powders modified in aqueous solutions of transition metal salts (PPTM). The insets in Fig. 1 present the typical examples of *F*(*E*) functions. Fig. 2

Fig. 1 shows Δ(*E*) dependences obtained for different PPTM. In order to minimize the influence of noise in the initial spectral data on the results of Δ(*E*) calculations, these data have been preliminarily smoothed using the Savitzky–Golay second-order filter with a 15-point window. The applied spectral method does not allow estimating the type of interband electron transition (direct or indirect); however, it allows assessing the data on energy-band-gap characteristics for the PPTM nanoparticles.

The Δ(*E*) plots for different PPTM possess two groups of peaks taking place in the obtained Δ(*E*) patterns. The 1st group is related to relatively high values of the photon energy and has higher intensity in comparison with another (patterns corresponding to the PPT modified with copper, nickel and zinc salts). The 2nd group appearing at lower photon energies might be characterized by higher intensity and has been observed for PPT modified with cobalt, iron and chromium salts.

Taking into account that a presence of multiple maxima in each group of peaks could be explained by structural features of different particles as well as their size [8], the data on energy-band-gap characteristics of the investigated group of products allow a meaningful choice of the PPTM type for its specific application in photocatalysis, opto-electronics, photovoltaics and different semiconductor or solid-state electrolytes based electronic devices.

## Figures and Tables

**Table 1:** Chemical compositions of the modified PPT powders represented in the oxide form (Me_x_O_y_ – oxide of the corresponding transition metal).

Salt used for the treatment	Content, wt%			
K_2_O	TiO_2_	Me_x_O_y_	SiO_2_
Copper (II) oxalate	8.7	80.2	9.9	1.2
Cobalt (II) oxalate	10.0	81.6	7.0	1.4
Iron (II) oxalate	10.5	80.9	7.4	1.2
Zinc fumarate	8.5	80.4	10.1	1.0
Chromium acetate	8.3	82.7	7.9	1.1
Nickel acetate	9.4	80.8	8.7	1.1
